# Ethyl 1′′-benzyl-1′-methyl-2′′-oxodi­spiro­[indeno­[1,2-*b*]quinoxaline-11,3′-pyrrolidine-2′,3′′-indoline]-4′-carboxyl­ate

**DOI:** 10.1107/S1600536813011525

**Published:** 2013-05-04

**Authors:** Piskala Subburaman Kannan, Srinu Lanka, Sathiah Thennarasu, Elumalai Govindan, Arunachalathevar SubbiahPandi

**Affiliations:** a Department of Physics, S.M.K. Fomra Institute of Technology, Thaiyur, Chennai 603 103, India; b Organic Chemistry Division, CSIR-Central Leather Research Institute, Adyar, Chennai 600 020, India; c Department of Physics, Presidency College (Autonomous), Chennai 600 005, India

## Abstract

In the title compound, C_36_H_30_N_4_O_3_, the quinoxaline–indene system is roughly planar, with a maximum deviation from the mean plane of 0.218 Å for the C atom shared with the central pyrrolidine ring. This latter ring forms dihedral angles of 84.54 (7) and 83.91 (8)° with the quinoxaline–indene system and the indole ring, respectively. The central pyrrolidine ring has an envelope conformation with the N atom as the flap, while the pyrrolidine and five-membered rings of the indole group adopt twisted conformation and envelope (with the C atom bearing the quinoxaline–indene system as the flap) conformations, respectively. In the crystal, mol­ecules are linked *via* weak C—H⋯N hydrogen bonds, forming a chain running along [100].

## Related literature

For details of the synthesis, see: Azizian *et al.* (2005 ▶). For uses of pyrrolidine and quinoxaline derivatives, see: Amal Raj *et al.* (2003 ▶); Zarranz *et al.* (2003 ▶). For a related structure, see: Srinivasan *et al.* (2012 ▶). For ring conformations, see: Cremer & Pople (1975 ▶).
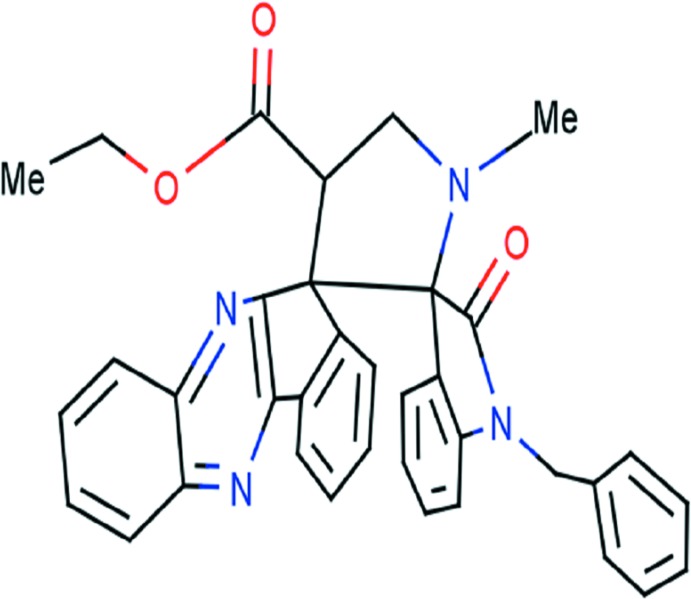


## Experimental

### 

#### Crystal data

C_36_H_30_N_4_O_3_
*M* *_r_* = 566.64
Triclinic, 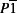

*a* = 11.1927 (2) Å
*b* = 11.4535 (3) Å
*c* = 12.1206 (3) Å
α = 87.637 (2)°
β = 86.048 (1)°
γ = 70.564 (2)°
*V* = 1461.50 (6) Å^3^
*Z* = 2
Mo *K*α radiation
μ = 0.08 mm^−1^
*T* = 293 K
0.35 × 0.30 × 0.25 mm

#### Data collection

Bruker APEXII CCD area detector diffractometer
Absorption correction: multi-scan (*SADABS*; Bruker, 2008 ▶) *T*_min_ = 0.971, *T*_max_ = 0.980
21785 measured reflections
5987 independent reflections
4956 reflections with *I* > 2σ(*I*)
*R*_int_ = 0.029

#### Refinement

*R*[*F* ^2^ > 2σ(*F* ^2^)] = 0.047
*wR*(*F* ^2^) = 0.134
*S* = 1.04
5987 reflections
388 parameters
H-atom parameters constrained
Δρ_max_ = 0.36 e Å^−3^
Δρ_min_ = −0.28 e Å^−3^

### 

Data collection: *APEX2* (Bruker, 2008 ▶); cell refinement: *SAINT* (Bruker, 2008 ▶); data reduction: *SAINT*; program(s) used to solve structure: *SHELXS97* (Sheldrick, 2008 ▶); program(s) used to refine structure: *SHELXL97* (Sheldrick, 2008 ▶); molecular graphics: *ORTEP-3 for Windows* (Farrugia, 2012 ▶); software used to prepare material for publication: *SHELXL97* and *PLATON* (Spek, 2009 ▶).

## Supplementary Material

Crystal structure: contains datablock(s) global, I. DOI: 10.1107/S1600536813011525/bg2503sup1.cif

Structure factors: contains datablock(s) I. DOI: 10.1107/S1600536813011525/bg2503Isup2.hkl

Additional supplementary materials:  crystallographic information; 3D view; checkCIF report

## Figures and Tables

**Table 1:** Hydrogen-bond geometry (Å, °)

*D*—H⋯*A*	*D*—H	H⋯*A*	*D*⋯*A*	*D*—H⋯*A*
C27—H27⋯N4^i^	0.93	2.60	3.446 (2)	152

Symmetry code: (i) 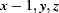.

**Fig. 1.**
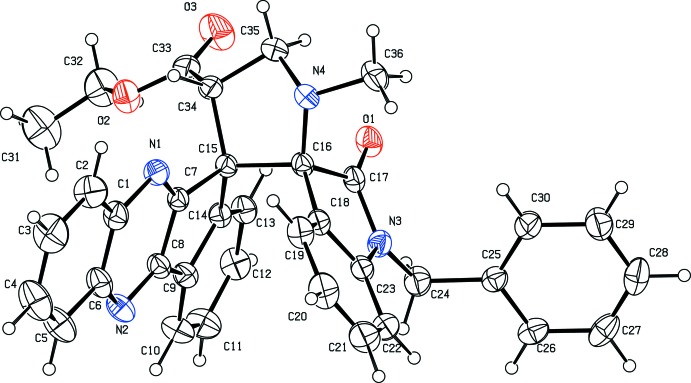
The molecular structure of the title compound with the atom numbering scheme. Displacement ellipsoids are drawn at the 30% probability level.

**Fig. 2.**
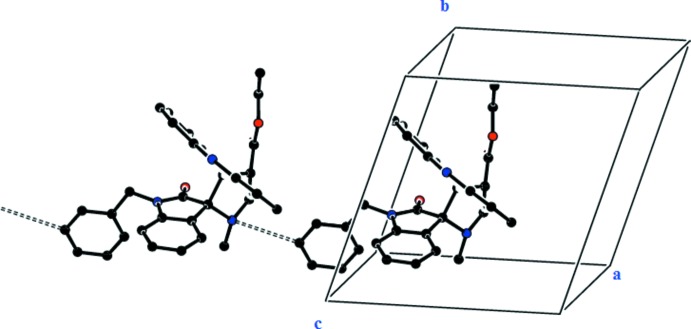
A packing viewed of the structure, projected down the c axis, showing the way in which the [100] chains are formed. Dashed lines represent the intermolecular C—H···N hydrogen bonds.

## supplementary crystallographic information

### Comment

Pyrrolidine derivatives are found to have anticonvulsant, antimicrobial and antifungal activities against various pathogens (Amal Raj *et al.*, 2003). Quinoxaline derivatives may show antibacterial, antiviral and anticancer properties (Zarranz *et al.*, 2003). As spiro pyrrolidine compounds are of interest due to their potential medicinal properties, we have undertaken the study of the three dimensional structure of the title compound C_36_H_30_N_4_O_3_, (I).

Fig 1 presents a molecular view of (I). The quinoxaline-indene system C1-C15/N1-N2), is essentially planar, with maximum deviation from the mean plane of 0.218Å for atom C15.

The central pyrrolidine ring (N4/C15-C16/C34-C35) forms dihedral angles of 84.54 (7) and 83.91 (8)° with the quinoxaline-indene and the (C16-C23/N3 indole groups, respectively. The central pyrrolidine ring is enveloped on N4 with puckering parameters q_2_ = 0.4000 (2) Å, φ = 359.20 (2)° (Cremer & Pople, 1975). The pyrrolidine in the indole group adopts a twisted conformation on C17-C16 with puckering parameters of q_2_ = 0.1265 (2) Å, φ = 51.90 (7)°, while the (C7-C9/C14-C15) five membered ring envelopes on C15 with puckering parameters q_2_ = 0.1135 (2) Å, φ = 322.40 (8)°.

In the crystal packing, molecules are linked via weak C-H···N intermolecular hydrogen bonds (Table 1) to form chains along [100], as shown in Fig.2.

### Experimental

A mixture of benzyl Isatin(0.25 mmol), sarcosine(0.3 mmol), ethyl indeno[1,2-b]quinoxalin-11-ylideneacetate(0.25 mmol) in ethanol was refluxed for 60 min (Azizian *et al.*, 2005). The progress of the reaction was followed by TLC. After completion, the solvent was removed under reduced pressure and the resulting crude product was subjected to column chromatography. The product was recrystallised from methanol. Single crystals suitable for X-ray diffraction were obtained by slow evaporation of a solution of the title compound in methanol at room temperature.

### Refinement

All H atoms were fixed geometrically and allowed to ride on their parent C atoms, with C—H distances fixed in the range 0.93–0.97 Å with *U*_iso_(H) = 1.5*U*_eq_(C) for methyl and 1.2*U*_eq_(C) for all other H atoms. The positions of methyl hydrogens were optimized rotationally.

### Crystal data

**Table:**

C_36_H_30_N_4_O_3_	*Z* = 2
*M**_r_* = 566.64	*F*(000) = 596
Triclinic, *P*1	*D*_x_ = 1.288 Mg m^−^^3^
Hall symbol: -P 1	Mo *K*α radiation, λ = 0.71073 Å
*a* = 11.1927 (2) Å	Cell parameters from 5987 reflections
*b* = 11.4535 (3) Å	θ = 1.7–26.4°
*c* = 12.1206 (3) Å	µ = 0.08 mm^−^^1^
α = 87.637 (2)°	*T* = 293 K
β = 86.048 (1)°	Block, colourless
γ = 70.564 (2)°	0.35 × 0.30 × 0.25 mm
*V* = 1461.50 (6) Å^3^	

### Data collection

**Table:**

Bruker APEXII CCD area detector diffractometer	5987 independent reflections
Radiation source: fine-focus sealed tube	4956 reflections with *I* > 2σ(*I*)
Graphite monochromator	*R*_int_ = 0.029
ω and φ scans	θ_max_ = 26.4°, θ_min_ = 1.7°
Absorption correction: multi-scan (*SADABS*; Bruker, 2008)	*h* = −13→13
*T*_min_ = 0.971, *T*_max_ = 0.980	*k* = −14→14
21785 measured reflections	*l* = −15→15

### Refinement

**Table:**

Refinement on *F*^2^	Primary atom site location: structure-invariant direct methods
Least-squares matrix: full	Secondary atom site location: difference Fourier map
*R*[*F*^2^ > 2σ(*F*^2^)] = 0.047	Hydrogen site location: inferred from neighbouring sites
*wR*(*F*^2^) = 0.134	H-atom parameters constrained
*S* = 1.04	*w* = 1/[σ^2^(*F*_o_^2^) + (0.0673*P*)^2^ + 0.3957*P*] where *P* = (*F*_o_^2^ + 2*F*_c_^2^)/3
5987 reflections	(Δ/σ)_max_ < 0.001
388 parameters	Δρ_max_ = 0.36 e Å^−^^3^
0 restraints	Δρ_min_ = −0.28 e Å^−^^3^

### Special details

**Table:**

Geometry. All esds (except the esd in the dihedral angle between two l.s. planes) are estimated using the full covariance matrix. The cell esds are taken into account individually in the estimation of esds in distances, angles and torsion angles; correlations between esds in cell parameters are only used when they are defined by crystal symmetry. An approximate (isotropic) treatment of cell esds is used for estimating esds involving l.s. planes.
Refinement. Refinement of F^2^ against ALL reflections. The weighted R-factor wR and goodness of fit S are based on F^2^, conventional R-factors R are based on F, with F set to zero for negative F^2^. The threshold expression of F^2^ > 2sigma(F^2^) is used only for calculating R-factors(gt) etc. and is not relevant to the choice of reflections for refinement. R-factors based on F^2^ are statistically about twice as large as those based on F, and R- factors based on ALL data will be even larger.

### Fractional atomic coordinates and isotropic or equivalent isotropic displacement parameters (Å^2^)

**Table:**

	*x*	*y*	*z*	*U*_iso_*/*U*_eq_	
C1	0.46495 (16)	0.33734 (15)	0.47540 (12)	0.0467 (4)	
C2	0.58578 (19)	0.2802 (2)	0.51621 (16)	0.0655 (5)	
H2	0.6504	0.2270	0.4717	0.079*	
C3	0.6081 (2)	0.3030 (2)	0.62131 (18)	0.0796 (6)	
H3	0.6886	0.2662	0.6475	0.096*	
C4	0.5112 (2)	0.3814 (2)	0.69003 (17)	0.0813 (7)	
H4	0.5276	0.3952	0.7617	0.098*	
C5	0.3943 (2)	0.4369 (2)	0.65289 (15)	0.0716 (6)	
H5	0.3306	0.4882	0.6994	0.086*	
C6	0.36772 (17)	0.41794 (15)	0.54397 (13)	0.0505 (4)	
C7	0.32937 (13)	0.36757 (13)	0.33956 (11)	0.0355 (3)	
C8	0.23311 (15)	0.45322 (13)	0.40719 (11)	0.0401 (3)	
C9	0.12264 (14)	0.50836 (13)	0.34192 (12)	0.0416 (3)	
C10	0.00864 (17)	0.59934 (16)	0.36960 (14)	0.0573 (4)	
H10	−0.0093	0.6301	0.4409	0.069*	
C11	−0.07808 (18)	0.64365 (17)	0.28945 (16)	0.0629 (5)	
H11	−0.1557	0.7039	0.3070	0.076*	
C12	−0.05019 (17)	0.59895 (16)	0.18324 (15)	0.0549 (4)	
H12	−0.1084	0.6315	0.1295	0.066*	
C13	0.06289 (15)	0.50652 (14)	0.15560 (13)	0.0451 (3)	
H13	0.0803	0.4767	0.0840	0.054*	
C14	0.15026 (13)	0.45856 (12)	0.23576 (11)	0.0373 (3)	
C15	0.27707 (13)	0.35239 (12)	0.23015 (11)	0.0345 (3)	
C16	0.25846 (13)	0.21964 (13)	0.22938 (11)	0.0362 (3)	
C17	0.13440 (14)	0.23436 (13)	0.17136 (12)	0.0396 (3)	
C18	0.22998 (15)	0.16963 (13)	0.34140 (11)	0.0398 (3)	
C19	0.30797 (18)	0.11634 (15)	0.42594 (13)	0.0519 (4)	
H19	0.3939	0.1070	0.4190	0.062*	
C20	0.2559 (2)	0.07688 (18)	0.52142 (15)	0.0671 (5)	
H20	0.3072	0.0415	0.5794	0.081*	
C21	0.1297 (2)	0.08966 (19)	0.53107 (15)	0.0697 (6)	
H21	0.0964	0.0642	0.5965	0.084*	
C22	0.0499 (2)	0.13956 (17)	0.44591 (15)	0.0597 (5)	
H22	−0.0355	0.1465	0.4524	0.072*	
C23	0.10286 (15)	0.17842 (13)	0.35109 (12)	0.0430 (3)	
C24	−0.08781 (15)	0.25705 (16)	0.23371 (16)	0.0538 (4)	
H24A	−0.1076	0.3166	0.1727	0.065*	
H24B	−0.1353	0.2980	0.2993	0.065*	
C25	−0.13298 (14)	0.15082 (14)	0.20920 (12)	0.0429 (3)	
C26	−0.25876 (15)	0.16256 (17)	0.23242 (13)	0.0505 (4)	
H26	−0.3132	0.2341	0.2656	0.061*	
C27	−0.30515 (17)	0.0699 (2)	0.20724 (15)	0.0612 (5)	
H27	−0.3901	0.0790	0.2237	0.073*	
C28	−0.2258 (2)	−0.0355 (2)	0.15803 (16)	0.0653 (5)	
H28	−0.2569	−0.0980	0.1409	0.078*	
C29	−0.1006 (2)	−0.04890 (18)	0.13409 (17)	0.0645 (5)	
H29	−0.0470	−0.1204	0.1004	0.077*	
C30	−0.05354 (16)	0.04371 (16)	0.15982 (15)	0.0551 (4)	
H30	0.0317	0.0338	0.1439	0.066*	
C31	0.2604 (3)	0.7740 (2)	0.0863 (2)	0.0978 (8)	
H31A	0.2272	0.8468	0.0407	0.147*	
H31B	0.3403	0.7718	0.1123	0.147*	
H31C	0.2018	0.7759	0.1484	0.147*	
C32	0.2783 (3)	0.6663 (2)	0.02305 (18)	0.0802 (7)	
H32A	0.3364	0.6647	−0.0405	0.096*	
H32B	0.1979	0.6682	−0.0036	0.096*	
C33	0.33704 (16)	0.44973 (16)	0.04707 (12)	0.0481 (4)	
C34	0.37562 (14)	0.34434 (14)	0.12994 (11)	0.0402 (3)	
H34	0.4544	0.3451	0.1601	0.048*	
C35	0.40247 (16)	0.21799 (15)	0.08010 (13)	0.0488 (4)	
H35A	0.3479	0.2227	0.0201	0.059*	
H35B	0.4903	0.1840	0.0526	0.059*	
C36	0.37646 (19)	0.02159 (16)	0.14274 (16)	0.0613 (5)	
H36A	0.4566	−0.0218	0.1055	0.092*	
H36B	0.3094	0.0299	0.0946	0.092*	
H36C	0.3645	−0.0239	0.2084	0.092*	
N1	0.44398 (12)	0.31244 (12)	0.36901 (10)	0.0431 (3)	
N2	0.24881 (14)	0.47965 (13)	0.50795 (11)	0.0516 (3)	
N3	0.04620 (12)	0.22332 (12)	0.25098 (11)	0.0445 (3)	
N4	0.37489 (12)	0.14388 (11)	0.17259 (10)	0.0438 (3)	
O1	0.11649 (12)	0.25347 (11)	0.07356 (9)	0.0526 (3)	
O2	0.32960 (13)	0.55571 (11)	0.09209 (9)	0.0587 (3)	
O3	0.31436 (17)	0.44125 (14)	−0.04700 (10)	0.0807 (5)	

### Atomic displacement parameters (Å^2^)

**Table:**

	*U*^11^	*U*^22^	*U*^33^	*U*^12^	*U*^13^	*U*^23^
C1	0.0535 (9)	0.0549 (9)	0.0381 (8)	−0.0249 (7)	−0.0133 (7)	0.0024 (7)
C2	0.0579 (11)	0.0865 (14)	0.0518 (10)	−0.0209 (10)	−0.0190 (8)	0.0026 (9)
C3	0.0773 (14)	0.1032 (17)	0.0632 (12)	−0.0309 (13)	−0.0386 (11)	0.0092 (12)
C4	0.1098 (18)	0.0864 (15)	0.0518 (11)	−0.0305 (13)	−0.0403 (12)	−0.0051 (10)
C5	0.0979 (16)	0.0701 (12)	0.0437 (10)	−0.0186 (11)	−0.0255 (10)	−0.0102 (9)
C6	0.0674 (11)	0.0508 (9)	0.0373 (8)	−0.0222 (8)	−0.0147 (7)	−0.0029 (7)
C7	0.0425 (8)	0.0384 (7)	0.0302 (6)	−0.0190 (6)	−0.0046 (5)	−0.0007 (5)
C8	0.0500 (8)	0.0402 (7)	0.0325 (7)	−0.0174 (6)	−0.0038 (6)	−0.0039 (6)
C9	0.0474 (8)	0.0398 (7)	0.0374 (7)	−0.0137 (6)	−0.0045 (6)	−0.0018 (6)
C10	0.0597 (11)	0.0547 (10)	0.0469 (9)	−0.0041 (8)	−0.0011 (8)	−0.0097 (7)
C11	0.0549 (10)	0.0562 (10)	0.0630 (11)	0.0021 (8)	−0.0072 (8)	−0.0030 (8)
C12	0.0526 (10)	0.0512 (9)	0.0565 (10)	−0.0093 (7)	−0.0183 (8)	0.0064 (7)
C13	0.0514 (9)	0.0459 (8)	0.0395 (8)	−0.0168 (7)	−0.0114 (7)	0.0018 (6)
C14	0.0423 (8)	0.0365 (7)	0.0356 (7)	−0.0159 (6)	−0.0051 (6)	0.0000 (5)
C15	0.0395 (7)	0.0383 (7)	0.0287 (6)	−0.0162 (6)	−0.0047 (5)	−0.0007 (5)
C16	0.0428 (8)	0.0379 (7)	0.0303 (6)	−0.0160 (6)	−0.0054 (6)	−0.0017 (5)
C17	0.0481 (8)	0.0393 (7)	0.0363 (7)	−0.0196 (6)	−0.0082 (6)	−0.0029 (6)
C18	0.0526 (9)	0.0355 (7)	0.0348 (7)	−0.0188 (6)	−0.0050 (6)	−0.0007 (5)
C19	0.0680 (11)	0.0472 (8)	0.0447 (9)	−0.0231 (8)	−0.0167 (8)	0.0081 (7)
C20	0.1049 (17)	0.0611 (11)	0.0441 (9)	−0.0379 (11)	−0.0202 (10)	0.0159 (8)
C21	0.1144 (18)	0.0666 (12)	0.0388 (9)	−0.0469 (12)	0.0051 (10)	0.0062 (8)
C22	0.0739 (12)	0.0631 (11)	0.0516 (10)	−0.0381 (9)	0.0122 (9)	−0.0067 (8)
C23	0.0560 (9)	0.0389 (7)	0.0383 (8)	−0.0212 (7)	−0.0004 (6)	−0.0047 (6)
C24	0.0418 (9)	0.0488 (9)	0.0697 (11)	−0.0120 (7)	−0.0065 (8)	−0.0077 (8)
C25	0.0386 (8)	0.0508 (8)	0.0404 (8)	−0.0163 (6)	−0.0052 (6)	0.0018 (6)
C26	0.0395 (8)	0.0666 (10)	0.0439 (8)	−0.0163 (7)	−0.0052 (6)	0.0082 (7)
C27	0.0486 (10)	0.0891 (14)	0.0572 (10)	−0.0384 (10)	−0.0167 (8)	0.0245 (10)
C28	0.0812 (14)	0.0735 (12)	0.0610 (11)	−0.0502 (11)	−0.0256 (10)	0.0169 (10)
C29	0.0744 (13)	0.0559 (10)	0.0683 (12)	−0.0273 (9)	−0.0049 (10)	−0.0083 (9)
C30	0.0450 (9)	0.0555 (9)	0.0671 (11)	−0.0199 (7)	0.0023 (8)	−0.0081 (8)
C31	0.139 (2)	0.0601 (13)	0.0865 (17)	−0.0203 (14)	−0.0283 (16)	0.0143 (12)
C32	0.124 (2)	0.0633 (12)	0.0583 (12)	−0.0387 (13)	−0.0150 (12)	0.0239 (10)
C33	0.0567 (10)	0.0596 (9)	0.0328 (7)	−0.0262 (8)	−0.0013 (6)	0.0016 (7)
C34	0.0431 (8)	0.0499 (8)	0.0313 (7)	−0.0204 (6)	−0.0014 (6)	−0.0024 (6)
C35	0.0544 (9)	0.0547 (9)	0.0387 (8)	−0.0206 (7)	0.0062 (7)	−0.0096 (7)
C36	0.0738 (12)	0.0445 (9)	0.0654 (11)	−0.0188 (8)	0.0016 (9)	−0.0164 (8)
N1	0.0431 (7)	0.0525 (7)	0.0361 (6)	−0.0181 (6)	−0.0076 (5)	−0.0014 (5)
N2	0.0643 (9)	0.0524 (8)	0.0358 (7)	−0.0144 (7)	−0.0091 (6)	−0.0082 (6)
N3	0.0431 (7)	0.0494 (7)	0.0458 (7)	−0.0210 (6)	−0.0059 (5)	−0.0015 (5)
N4	0.0477 (7)	0.0404 (6)	0.0431 (7)	−0.0139 (5)	0.0012 (5)	−0.0092 (5)
O1	0.0673 (7)	0.0607 (7)	0.0368 (6)	−0.0282 (6)	−0.0168 (5)	0.0008 (5)
O2	0.0900 (9)	0.0532 (7)	0.0422 (6)	−0.0356 (6)	−0.0133 (6)	0.0103 (5)
O3	0.1283 (13)	0.0852 (10)	0.0349 (6)	−0.0421 (9)	−0.0181 (7)	0.0060 (6)

### Geometric parameters (Å, º)

**Table:**

C1—N1	1.3842 (19)	C20—H20	0.9300
C1—C2	1.407 (2)	C21—C22	1.388 (3)
C1—C6	1.413 (2)	C21—H21	0.9300
C2—C3	1.367 (3)	C22—C23	1.382 (2)
C2—H2	0.9300	C22—H22	0.9300
C3—C4	1.402 (3)	C23—N3	1.406 (2)
C3—H3	0.9300	C24—N3	1.447 (2)
C4—C5	1.351 (3)	C24—C25	1.511 (2)
C4—H4	0.9300	C24—H24A	0.9700
C5—C6	1.414 (2)	C24—H24B	0.9700
C5—H5	0.9300	C25—C26	1.379 (2)
C6—N2	1.375 (2)	C25—C30	1.384 (2)
C7—N1	1.2950 (19)	C26—C27	1.380 (3)
C7—C8	1.427 (2)	C26—H26	0.9300
C7—C15	1.5249 (17)	C27—C28	1.370 (3)
C8—N2	1.3075 (18)	C27—H27	0.9300
C8—C9	1.459 (2)	C28—C29	1.369 (3)
C9—C10	1.383 (2)	C28—H28	0.9300
C9—C14	1.402 (2)	C29—C30	1.387 (2)
C10—C11	1.380 (3)	C29—H29	0.9300
C10—H10	0.9300	C30—H30	0.9300
C11—C12	1.382 (3)	C31—C32	1.430 (3)
C11—H11	0.9300	C31—H31A	0.9600
C12—C13	1.383 (2)	C31—H31B	0.9600
C12—H12	0.9300	C31—H31C	0.9600
C13—C14	1.390 (2)	C32—O2	1.459 (2)
C13—H13	0.9300	C32—H32A	0.9700
C14—C15	1.5306 (19)	C32—H32B	0.9700
C15—C34	1.5677 (19)	C33—O3	1.1997 (19)
C15—C16	1.6023 (18)	C33—O2	1.326 (2)
C16—N4	1.4485 (19)	C33—C34	1.505 (2)
C16—C18	1.5069 (19)	C34—C35	1.519 (2)
C16—C17	1.5556 (19)	C34—H34	0.9800
C17—O1	1.2146 (17)	C35—N4	1.456 (2)
C17—N3	1.3651 (19)	C35—H35A	0.9700
C18—C19	1.379 (2)	C35—H35B	0.9700
C18—C23	1.390 (2)	C36—N4	1.455 (2)
C19—C20	1.386 (3)	C36—H36A	0.9600
C19—H19	0.9300	C36—H36B	0.9600
C20—C21	1.369 (3)	C36—H36C	0.9600
			
N1—C1—C2	119.04 (16)	C21—C22—H22	121.4
N1—C1—C6	121.56 (14)	C22—C23—C18	121.71 (15)
C2—C1—C6	119.40 (15)	C22—C23—N3	128.49 (16)
C3—C2—C1	119.9 (2)	C18—C23—N3	109.68 (13)
C3—C2—H2	120.0	N3—C24—C25	115.36 (13)
C1—C2—H2	120.0	N3—C24—H24A	108.4
C2—C3—C4	120.76 (19)	C25—C24—H24A	108.4
C2—C3—H3	119.6	N3—C24—H24B	108.4
C4—C3—H3	119.6	C25—C24—H24B	108.4
C5—C4—C3	120.43 (17)	H24A—C24—H24B	107.5
C5—C4—H4	119.8	C26—C25—C30	118.58 (15)
C3—C4—H4	119.8	C26—C25—C24	119.15 (15)
C4—C5—C6	120.7 (2)	C30—C25—C24	122.21 (14)
C4—C5—H5	119.7	C25—C26—C27	121.06 (17)
C6—C5—H5	119.7	C25—C26—H26	119.5
N2—C6—C1	122.27 (13)	C27—C26—H26	119.5
N2—C6—C5	118.93 (17)	C28—C27—C26	119.89 (16)
C1—C6—C5	118.79 (16)	C28—C27—H27	120.1
N1—C7—C8	123.68 (13)	C26—C27—H27	120.1
N1—C7—C15	126.17 (13)	C29—C28—C27	119.96 (17)
C8—C7—C15	110.16 (12)	C29—C28—H28	120.0
N2—C8—C7	123.74 (14)	C27—C28—H28	120.0
N2—C8—C9	127.91 (14)	C28—C29—C30	120.32 (18)
C7—C8—C9	108.24 (12)	C28—C29—H29	119.8
C10—C9—C14	121.55 (14)	C30—C29—H29	119.8
C10—C9—C8	129.74 (14)	C25—C30—C29	120.19 (16)
C14—C9—C8	108.63 (13)	C25—C30—H30	119.9
C11—C10—C9	118.74 (16)	C29—C30—H30	119.9
C11—C10—H10	120.6	C32—C31—H31A	109.5
C9—C10—H10	120.6	C32—C31—H31B	109.5
C10—C11—C12	120.38 (16)	H31A—C31—H31B	109.5
C10—C11—H11	119.8	C32—C31—H31C	109.5
C12—C11—H11	119.8	H31A—C31—H31C	109.5
C11—C12—C13	121.07 (15)	H31B—C31—H31C	109.5
C11—C12—H12	119.5	C31—C32—O2	109.46 (18)
C13—C12—H12	119.5	C31—C32—H32A	109.8
C12—C13—C14	119.45 (14)	O2—C32—H32A	109.8
C12—C13—H13	120.3	C31—C32—H32B	109.8
C14—C13—H13	120.3	O2—C32—H32B	109.8
C13—C14—C9	118.73 (14)	H32A—C32—H32B	108.2
C13—C14—C15	130.38 (13)	O3—C33—O2	123.62 (16)
C9—C14—C15	110.87 (12)	O3—C33—C34	125.49 (16)
C7—C15—C14	100.73 (11)	O2—C33—C34	110.89 (12)
C7—C15—C34	112.04 (11)	C33—C34—C35	113.57 (12)
C14—C15—C34	120.22 (11)	C33—C34—C15	114.86 (12)
C7—C15—C16	109.35 (10)	C35—C34—C15	106.14 (11)
C14—C15—C16	112.16 (11)	C33—C34—H34	107.3
C34—C15—C16	102.35 (10)	C35—C34—H34	107.3
N4—C16—C18	114.48 (12)	C15—C34—H34	107.3
N4—C16—C17	115.63 (11)	N4—C35—C34	103.40 (11)
C18—C16—C17	101.08 (11)	N4—C35—H35A	111.1
N4—C16—C15	102.63 (11)	C34—C35—H35A	111.1
C18—C16—C15	115.02 (11)	N4—C35—H35B	111.1
C17—C16—C15	108.36 (11)	C34—C35—H35B	111.1
O1—C17—N3	125.16 (14)	H35A—C35—H35B	109.0
O1—C17—C16	127.30 (14)	N4—C36—H36A	109.5
N3—C17—C16	107.53 (11)	N4—C36—H36B	109.5
C19—C18—C23	119.96 (14)	H36A—C36—H36B	109.5
C19—C18—C16	130.95 (15)	N4—C36—H36C	109.5
C23—C18—C16	109.03 (12)	H36A—C36—H36C	109.5
C18—C19—C20	118.79 (17)	H36B—C36—H36C	109.5
C18—C19—H19	120.6	C7—N1—C1	114.53 (13)
C20—C19—H19	120.6	C8—N2—C6	114.07 (14)
C21—C20—C19	120.53 (17)	C17—N3—C23	110.92 (12)
C21—C20—H20	119.7	C17—N3—C24	123.73 (13)
C19—C20—H20	119.7	C23—N3—C24	125.33 (14)
C20—C21—C22	121.83 (17)	C16—N4—C36	115.10 (13)
C20—C21—H21	119.1	C16—N4—C35	107.01 (12)
C22—C21—H21	119.1	C36—N4—C35	114.35 (13)
C23—C22—C21	117.12 (18)	C33—O2—C32	115.43 (14)
C23—C22—H22	121.4		
			
N1—C1—C2—C3	−179.60 (18)	C23—C18—C19—C20	−2.6 (2)
C6—C1—C2—C3	0.0 (3)	C16—C18—C19—C20	−179.42 (15)
C1—C2—C3—C4	1.1 (4)	C18—C19—C20—C21	0.6 (3)
C2—C3—C4—C5	−0.8 (4)	C19—C20—C21—C22	1.4 (3)
C3—C4—C5—C6	−0.5 (4)	C20—C21—C22—C23	−1.3 (3)
N1—C1—C6—N2	−2.5 (3)	C21—C22—C23—C18	−0.8 (2)
C2—C1—C6—N2	177.92 (16)	C21—C22—C23—N3	174.94 (16)
N1—C1—C6—C5	178.32 (17)	C19—C18—C23—C22	2.7 (2)
C2—C1—C6—C5	−1.2 (3)	C16—C18—C23—C22	−179.78 (14)
C4—C5—C6—N2	−177.67 (19)	C19—C18—C23—N3	−173.72 (13)
C4—C5—C6—C1	1.5 (3)	C16—C18—C23—N3	3.76 (16)
N1—C7—C8—N2	−3.8 (2)	N3—C24—C25—C26	155.66 (15)
C15—C7—C8—N2	176.38 (14)	N3—C24—C25—C30	−27.1 (2)
N1—C7—C8—C9	172.61 (13)	C30—C25—C26—C27	0.0 (2)
C15—C7—C8—C9	−7.16 (16)	C24—C25—C26—C27	177.35 (15)
N2—C8—C9—C10	−0.8 (3)	C25—C26—C27—C28	−0.2 (2)
C7—C8—C9—C10	−177.05 (17)	C26—C27—C28—C29	0.1 (3)
N2—C8—C9—C14	175.83 (15)	C27—C28—C29—C30	0.2 (3)
C7—C8—C9—C14	−0.44 (16)	C26—C25—C30—C29	0.3 (3)
C14—C9—C10—C11	−1.6 (3)	C24—C25—C30—C29	−176.91 (17)
C8—C9—C10—C11	174.65 (17)	C28—C29—C30—C25	−0.5 (3)
C9—C10—C11—C12	−1.0 (3)	O3—C33—C34—C35	8.2 (2)
C10—C11—C12—C13	2.0 (3)	O2—C33—C34—C35	−172.55 (14)
C11—C12—C13—C14	−0.5 (3)	O3—C33—C34—C15	−114.25 (19)
C12—C13—C14—C9	−2.0 (2)	O2—C33—C34—C15	65.02 (17)
C12—C13—C14—C15	175.86 (15)	C7—C15—C34—C33	−115.27 (14)
C10—C9—C14—C13	3.1 (2)	C14—C15—C34—C33	2.65 (18)
C8—C9—C14—C13	−173.87 (13)	C16—C15—C34—C33	127.70 (12)
C10—C9—C14—C15	−175.17 (15)	C7—C15—C34—C35	118.37 (13)
C8—C9—C14—C15	7.88 (16)	C14—C15—C34—C35	−123.71 (13)
N1—C7—C15—C14	−168.74 (13)	C16—C15—C34—C35	1.34 (14)
C8—C7—C15—C14	11.03 (14)	C33—C34—C35—N4	−152.74 (13)
N1—C7—C15—C34	−39.74 (19)	C15—C34—C35—N4	−25.61 (15)
C8—C7—C15—C34	140.03 (12)	C8—C7—N1—C1	3.7 (2)
N1—C7—C15—C16	73.01 (17)	C15—C7—N1—C1	−176.57 (13)
C8—C7—C15—C16	−107.22 (13)	C2—C1—N1—C7	178.85 (15)
C13—C14—C15—C7	170.60 (14)	C6—C1—N1—C7	−0.7 (2)
C9—C14—C15—C7	−11.42 (14)	C7—C8—N2—C6	0.4 (2)
C13—C14—C15—C34	47.1 (2)	C9—C8—N2—C6	−175.31 (15)
C9—C14—C15—C34	−134.95 (13)	C1—C6—N2—C8	2.5 (2)
C13—C14—C15—C16	−73.22 (18)	C5—C6—N2—C8	−178.34 (16)
C9—C14—C15—C16	104.77 (13)	O1—C17—N3—C23	168.75 (14)
C7—C15—C16—N4	−95.51 (12)	C16—C17—N3—C23	−11.93 (15)
C14—C15—C16—N4	153.63 (11)	O1—C17—N3—C24	−12.8 (2)
C34—C15—C16—N4	23.44 (13)	C16—C17—N3—C24	166.53 (13)
C7—C15—C16—C18	29.47 (16)	C22—C23—N3—C17	−170.70 (15)
C14—C15—C16—C18	−81.39 (14)	C18—C23—N3—C17	5.45 (16)
C34—C15—C16—C18	148.41 (12)	C22—C23—N3—C24	10.9 (2)
C7—C15—C16—C17	141.70 (12)	C18—C23—N3—C24	−172.98 (13)
C14—C15—C16—C17	30.85 (15)	C25—C24—N3—C17	98.76 (18)
C34—C15—C16—C17	−99.35 (12)	C25—C24—N3—C23	−83.00 (19)
N4—C16—C17—O1	−43.3 (2)	C18—C16—N4—C36	64.59 (17)
C18—C16—C17—O1	−167.54 (14)	C17—C16—N4—C36	−52.33 (18)
C15—C16—C17—O1	71.18 (18)	C15—C16—N4—C36	−170.08 (13)
N4—C16—C17—N3	137.37 (12)	C18—C16—N4—C35	−167.14 (12)
C18—C16—C17—N3	13.15 (14)	C17—C16—N4—C35	75.94 (15)
C15—C16—C17—N3	−108.12 (12)	C15—C16—N4—C35	−41.81 (13)
N4—C16—C18—C19	42.1 (2)	C34—C35—N4—C16	43.20 (15)
C17—C16—C18—C19	167.08 (15)	C34—C35—N4—C36	171.90 (13)
C15—C16—C18—C19	−76.45 (19)	O3—C33—O2—C32	6.3 (3)
N4—C16—C18—C23	−135.02 (13)	C34—C33—O2—C32	−172.97 (16)
C17—C16—C18—C23	−10.03 (14)	C31—C32—O2—C33	172.6 (2)
C15—C16—C18—C23	106.44 (13)		

### Hydrogen-bond geometry (Å, º)

**Table:**

*D*—H···*A*	*D*—H	H···*A*	*D*···*A*	*D*—H···*A*
C27—H27···N4^i^	0.93	2.60	3.446 (2)	152

Symmetry code: (i) *x*−1, *y*, *z*.
